# Designing accountability measures for health professionals: results from a community-based micro-credential: case study on Indigenous cultural safety

**DOI:** 10.1186/s12889-023-15721-9

**Published:** 2023-05-12

**Authors:** Angela Mashford-Pringle, Sharon Tan, Sterling Stutz, Gabriel Tjong

**Affiliations:** 1 Waakebiness-Bryce Institute for Indigenous Health, University of Toronto, 155 College Street, Toronto, ON M5T 3M7 Canada; 2 Dalla Lana School of Public Health, University of Toronto, Toronto, Canada

**Keywords:** Indigenous peoples, Cultural Safety, Health administration, Performance review, Evaluation tool, Public health, Implementation science

## Abstract

**Background:**

There is a widespread commitment to implementing anti-Indigenous racism with health organizations in Canada by introducing cultural safety staff training. In partnership with a public health unit in Ontario, Canada, we developed an evaluation tool to assess the performance of staff who completed an online Indigenous cultural safety education course.

**Aims:**

To develop an accountability checklist that could be used for annual employee performance reviews to assess the use and level of knowledge received in professional cultural safety training.

**Intervention:**

We co-created a professional development accountability checklist. Five areas of interest were identified: terminology, knowledge, awareness, skills, and behaviours. The checklist comprises of 37 indicators linked to our community collaborators’ intended goals as defined in our partnership agreement.

**Outcomes:**

The Indigenous Cultural Safety Evaluation Checklist (ICSEC) was shared with public health managers to use during regularly scheduled staff performance evaluations. The public health managers provided feedback on the design, checklist items, and useability of the ICSEC. The pilot of the checklist is in the preliminary stage and data is unavailable about effectiveness.

**Implications:**

Accountability tools are important to sustain the long-term effects of cultural safety education and prioritize the wellbeing of Indigenous communities. Our experience can provide guidance to health professionals in creating and measuring the efficacy of Indigenous cultural safety education to foster an anti-racist work culture as well as improved health outcomes among Indigenous communities.

**Supplementary Information:**

The online version contains supplementary material available at 10.1186/s12889-023-15721-9.

## Background

Experiences in health care among patients are influenced by the knowledge base and worldviews of health practitioners. Indigenous patients are more likely to experience higher number of racist encounters when accessing healthcare and social services [1–7], which in many ways has been exacerbated during the COVID-19 pandemic [8]. In Canada, Indigenous Peoples refer to First Nations, Inuit, and Métis. The Truth and Reconciliation Commission of Canada calls upon all health care organizations to provide mandatory cultural safety training to their health professionals to reduce racism, discrimination, and harm to Indigenous peoples when they seek care [9]. The need to learn how to better serve Indigenous patients is also upheld in the province of Ontario’s Ministry of Health and Long-Term Care guideline, Relationships with Indigenous Communities Guidelines, where they recommend that health professionals learn about local Indigenous peoples and build true and authentic relationships with Indigenous peoples, communities and organizations with the goal of improving relations and reducing racism and microaggressions reported by Indigenous peoples who access healthcare in the province [10]. Hiscock et al. [11] argue that Indigenous patient navigators would assist Indigenous patients with navigating the healthcare system as well as being a knowledge broker between health professionals and Indigenous peoples. The need to ensure cultural safety in accessing health care services is paramount; and yet, it is unclear how and if these trainings have been provided and what accountability mechanisms exist to ensure that staff participate meaningfully and incorporate the information into their daily health care practice.

The literature suggests that there is a dearth of published accountability measures and tools that are intended to hold staff accountable for implementing their cultural safety training in organizational settings, especially in Canada. Most of the cultural safety literature is from the United States and Australia [12] and may not directly reflect the Canadian context. A systematic review of reviews conducted by Troung et al. [13] looking at cultural competency interventions in healthcare found that a majority of studies were from the United States. A lack of studies evaluating cultural safety interventions and tools to assess these interventions is also noted in the literature [12–15]. Furthermore, there is a lack of studies conducting evaluations in organizational settings [16, 17]. A grey literature search reveals a lack of measures and tools specific to Indigenous cultural safety work in Canada. The resources and tools that have been developed we have found have either not been developed specific to the Canadian context or are relevant to cultural safety in general but are not specific to providing health and social services to and with Indigenous peoples [18, 19]. An environmental scan of available Indigenous cultural safety trainings in Ontario, Canada revealed that only three trainings used accountability measures to monitor and ensure participant completion of the training but the researchers did not find any measures to monitor longitudinal uptake of course content [17]. Our research provides a detailed account of how a tailored evaluation tool for a cohort of health practitioners in Ontario, Canada was developed to assess staff performance and measure accountability. This tool can be used longitudinally after course completion to monitor and support staff’s development of Indigenous cultural safety competencies.

### Cultural safety

Cultural safety can be viewed on a continuum that moves from cultural awareness to cultural sensitivity to cultural competency and ending with cultural safety. Cultural awareness refers to the acknowledgement of cultural differences [20]. Cultural sensitivity is respecting these cultural differences [20]. Cultural competency refers to a set of skills required to work in cross-cultural settings and can include a services provider’s knowledge of and attitudes towards their clients [20, 21]. Unlike cultural competency, which is defined by the service provider, cultural safety is defined by the client and requires that a service provider self-reflects about power imbalances and harmful biases they may hold [22]. Cultural safety, within the context of healthcare delivery, requires healthcare providers to consider the broader social, political, and historical difference contexts of patients including the consequences of racism and discrimination [20]. Cultural safety is inherently reflexive as a practice, requiring the health care provider to not only operate with a sufficient level of cultural competency towards their patients, but also to identify and understand their own sets of values and norms and how a healthcare provider’s cultural context might influence how their patient received healthcare service [20].

Unlike cultural awareness training, it is not sufficient to simply provide staff with an overview of what cultural safety is and expect that health care providers can now integrate the content into their practice. Cultural safety is a journey which healthcare providers must choose to accept and feel personally committed to developing, as well as being held accountable by their accreditation bodies and employers. In addition to mandating the completion of cultural safety trainings, workplaces can also incentivize the integration of cultural safety into healthcare practice by including it into the organization’s performance management evaluations [22] and requiring competency in cultural safety to qualify for promotions.

### What do we mean by accountability?

Each person is accountable for and to different peoples, organizations, and bodies based on the context the person is situated within. In the context of cultural safety, organizations are often held accountable to foster environments that prioritize the worldviews, values, and needs of Indigenous communities, delivering healthcare equitably, and incorporating regular reviews of staff [9, 22–24]. Within healthcare, accountability may involve staff and organizational evaluations to ensure processes and goals are being attained; these are commonly published in annual reports to enhance transparency and public knowledge [25]. Accountability measures are increasingly important as they assist in monitoring staff for culturally safe, appropriate, and patient-centred care. Within the contexts of Indigenous health and wellness, the idea of accountability is related to targeting the impacts of colonialism, including the mistrust of institutions, particularly the healthcare system and governments [26].

**Fig. 1:**
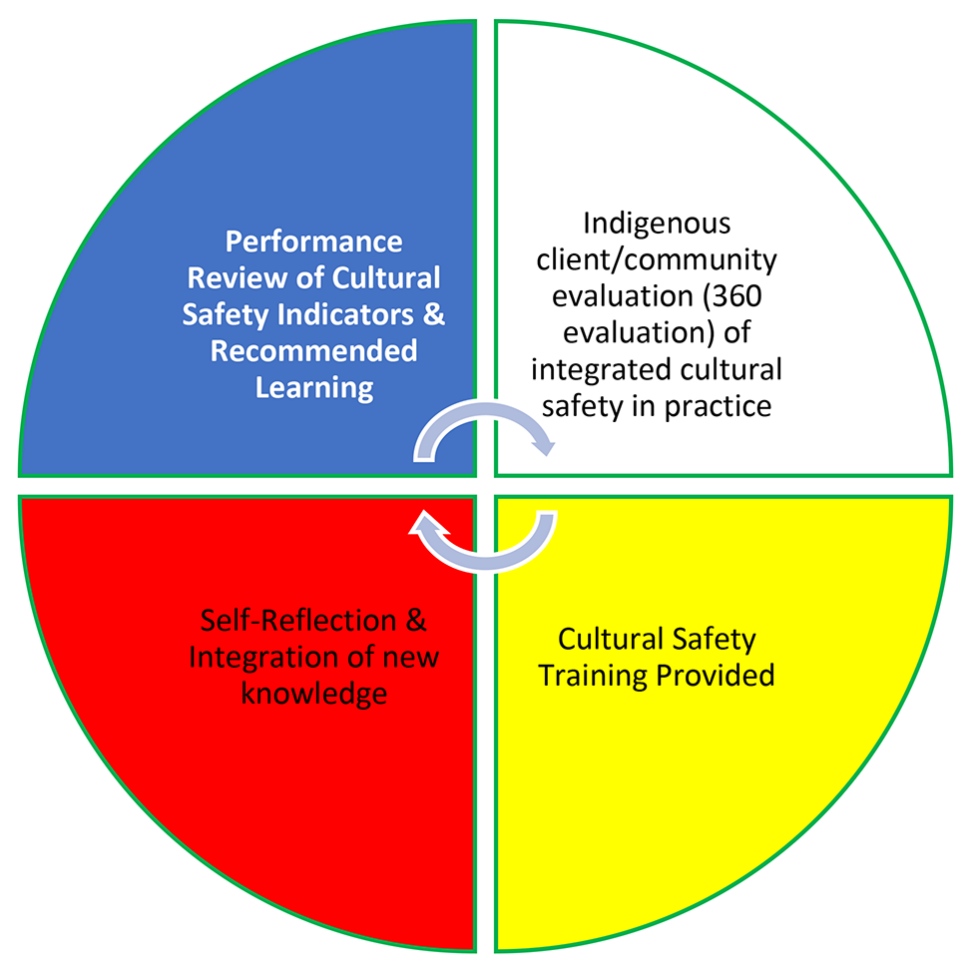
Relationships involved in staff evaluation

Practicing accountability can take different forms, but a key priority is ensuring the voices of Indigenous Peoples lead the way of defining what is culturally safe care, and that healthcare organizations and authorities are held accountable for delivering such care [27]. Some Indigenous Peoples regard accountability as a responsibility for the safety and wellbeing of individuals and community informed by collective values, community health and well-being, harm prevention, and dismantling of power imbalance in structures and organizations [28]. Indigenous communities see accountability as individual, familial, community, nation and all in creation, therefore health professionals must examine their accountability for culturally safe practice to Indigenous person(s) and community(s) as well as to the general public as a responsible provider and human being [29–31]. In the Royal Commission on Aboriginal People (RCAP) report [32], accountability was a key theme and guiding principle throughout, expressing that there is an important accountability to Indigenous peoples with all related to their well-being (p. 655). Subsequently, the Truth and Reconciliation Commission (TRC) of Canada’s 57th Call to Action recommended the provision of education on Indigenous rights, law, and residential schools, alongside skills-based training in cultural competency and anti-racism among federal, provincial, territorial, and municipal public servants [9]. As stated in the TRC’s Final Report [33], accountability in this context goes beyond an apology and rather, must encompass mutual respect and meaningful dialogue that aims for coexistence (p. 217–218). Extending these statements, non-Indigenous organizations, like public health units with non-Indigenous leadership, must be held accountable for providing adequate and appropriate cultural safety training and then determining if the skills gained are being used and the impact of culturally safe staff on the wellbeing of Indigenous peoples. In our project, accountability takes on different forms and relationships. Figure 1 describes the relationships and power dynamics involved in using the staff evaluation tool. Drawing upon Wilson [34], relationality and relational accountability exist within the same realm and manifest through our physical practices, methodologies, and ethics.

The motivation to be held accountable should shift from one rooted in colonial, patriarchal ideas to one that embraces Indigenous peoples, worldviews, and cultures which is centred on optimizing community health. Accountability, in this way, is one of relationship and invites public health staff to engage in relational accountability with the Indigenous communities and Nations whom they serve [27, 34]. Health professionals who embody personal and professional accountability for integrating the skills of cultural safety also assist Indigenous peoples, communities and Nations to heal from colonial violence while uplifting and being allies for the return to self-determination and governance for Indigenous individuals, families, communities and Nations [4]; it is through embodying and valuing Indigenous peoples, worldviews, and knowledges that decentres and decolonizes hierarchy in favour of true and authentic relationships who subscribe to the 7 Grandfather teachings of love, respect, humility, bravery, truth, honesty and wisdom. The 7 Grandfather teachings are sacred teachings for the Anishinaabe peoples in Ontario and Quebec to provide ways of living in harmony with all in creation (humans, animals, birds, insects, plants, trees, water, land, etc.) [35].

Cultural safety is often understood from a theoretical perspective through the Transtheoretical Model of Behaviour Change, also known as Stages of Change [23, 36, 37]. Although this is a model created by Western scholars, it is fitting and relevant to the epistemologies and worldviews of the target audience of cultural safety training, often being non-Indigenous peoples [37]. According to the Transtheoretical Model of Behavior Change, people must move through six stages to obtain change: precontemplation, contemplation, preparation, action, maintenance, and termination [38, 39]. Individuals go through this process in a cyclical manner as it may take many attempts to achieve change [40]. In the context of cultural safety and our project, precontemplation may not be as related as the broader non-Indigenous community is aware that cultural safety is needed and individuals are ready to take action to improve health and wellbeing [10, 37]. Individuals must contemplate the change to be made, even if it is mandated, then determine the course of action, and start the action. They may or may not enter a relapse stage before they require some re-learning and reinforcement of the knowledge that helps to change their behaviour or using the Social Norms theory [41, 42], they are confronted by the perceived norms (i.e., it’s okay to say/do microaggressions or racist remarks/behaviours) rather than reflecting the actual norm in society which shows more harmony and social justice. The misperception between these concepts is a space of discomfort that Indigenous cultural safety brings about before a person can self-reflect toward personal change. Operationalizing accountability through the development of tools ensures the delivery of person-centred care by staff who may unintentionally or intentionally harm, or who hold power in the patient-provider relationship [31].

## Aims

In collaboration with a public health unit in Ontario, we developed the Indigenous Cultural Safety Evaluation Checklist (ICSEC) to assess the performance of staff after the completion of an online Indigenous cultural safety education course. The objective is to provide a tangible tool for public health management to incentivize the implementation of cultural safety in staff’s professional practice and evaluate the program’s uptake. The ICSEC is an accountability checklist that can be used to assess the use and level of knowledge received in professional cultural safety training such as the cultural safety micro-credential [authors’ papers blinded]. The results of the forthcoming evaluation can support each organization and accreditation body (i.e., physicians’ college) in determining the implications (e.g., administrative, financial, social) of implementing Indigenous cultural safety training.

## Methods

This project incorporated a community-based approach grounded in Indigenous research principles and protocols to ensure activities were carried out in alignment with Indigenous ways of doing and knowing [34, 43, 44]. A partnership agreement with the partner agency was established at the beginning of the project to delineate the partner’s role and responsibilities. The principles of reciprocity, respect, and sharing guided our engagements, design, implementation, and evaluation [45].

In developing the online training program, the research team first connected with First Nations and Métis Elders, local First Nations and Métis community members, health professionals, and Indigenous scholars to design and develop community-relevant course content. The content developed was based on the local Indigenous communities’ interactions with the public health staff and the perceptions of the Indigenous communities about what stereotypes, myths, and facts needed to be addressed to have culturally safe care when working with public health professionals. Discussions with health professionals and Indigenous scholars were relevant for ensuring the content included was both relevant to the learning needs of staff working in public health and healthcare settings and that the content reflected current literature and best practices related to Indigenous cultural safety pedagogy. A three-hour micro-credential was created and included the administration of both a pre- and post- survey where participants were required to self-assess their knowledge and attitudes. Throughout the self-paced micro-credential, participants were required to complete related readings, videos, and quizzes [46].

The public health unit requested the development of a tool to evaluate the performance of staff regarding Indigenous cultural safety skills, so the research team engaged with the senior managers at the public health unit over a series of video conference calls to ensure the staff evaluation tool (ICSEC) was relevant and useful for local contexts including required competencies for working with Indigenous peoples and communities for public health staff. As we brought our ideas for the evaluation tool to the health unit senior leadership, we discussed the evaluation tool development and answered clarifying questions about the proposed indicators which, after clarifications, the health unit agreed with. The research team met with Indigenous and public health community partners several times in November and December of 2020 to gain a fuller understanding of the purpose of the tool, and to outline the organizational goals and guiding values and frameworks of the tool. This included an examination of organizational reports and governmental policies, which guide the present activities of the public health unit.

The research team developed the cultural safety evaluation tool as a checklist for use by senior managers to monitor and evaluate staff competency in practicing cultural safety in interacting with Indigenous peoples. The tool was based on the micro-credential curriculum as well as input from the senior managers at the public health unit and the Elders who helped design the micro-credential. Senior managers, as defined by the public health unit’s own organizational chart, will use the tool to assess how and if staff are making modifications to their work responsibilities, especially as it relates to Indigenous community members and clients. Managers were provided a copy of the tool to review in November 2021 and were invited to attend a virtual talking circle in December 2021 to discuss the strengths and challenges when using the tool as well as future directions.

As this project was completed during the COVID-19 pandemic, public health measures restricted in-person engagements with community partners. However, the research team and community partners mutually agreed upon leveraging virtual means, such as video conference calls and web applications to maintain consistent lines of communication and ongoing transparency. Since this project involved a public health unit that had priorities to respond to pressing issues related to COVID-19, there were some challenges in engaging in live, synchronous communication. As our focus was on ensuring this project produced more benefits than strain for the public health unit, we accommodated the needs of our partners by meeting in alignment with their schedules.

## Results

### The evaluation checklist

After consulting with the main users of the Indigenous Cultural Safety Evaluation Checklist (ICSEC or “the tool”), a checklist containing five components was developed. The components are: (1) terminology; (2) knowledge; (3) awareness; (4) skills; (5) behaviours (See Table 1 for objective of each component). The tool offers an uncomplicated way of assessing the process and progress of each staff’s journey towards cultural safety. Each component contains 5 to 10 measures, of which some are further divided and simplified into specific indicators. For example, “Appropriate use of terminology” is broken down into four forms of communication: orally in internal settings, in online internal communications, orally with Indigenous communities, and in online communications with Indigenous communities. Measures of each indicator can be rated as “Not in place”, “In progress”, “Completed/In place”, “Not applicable”, and “Don’t know”.

**Table 1:** Overview of Components in Staff Evaluation Checklist

**Components in Staff Performance Checklist**
**Terminology** - This component assesses the staff member’s knowledge about appropriate use of Indigenous health terminology and land acknowledgments. **Knowledge** - This component assesses the staff member’s knowledge of Indigenous cultural protocols and engagement, significance of traditional tobacco, Indigenous worldviews, Indigenous health concepts, and the impacts of colonization, **Awareness** - This component assesses the staff member’s self-awareness of concepts related to their social positionality including power and privilege. **Skills** - This component assesses the staff member’s skills in appropriate, respectful, and confident engagement with Indigenous communities given rising local contexts, as it relates to their job responsibilities. **Behaviours** - This component assesses the staff member’s ability to address community needs, foster long-term relations, and advocate on behalf of Indigenous communities.

In designing the tool, we incorporated Indigenous approaches to evaluation [43, 47–49], knowledge of the Indigenous cultural safety continuum [20], and the Transtheoretical model [16]. We had discussions with First Nations and Métis Elders, community members and organizations to determine what would be indicators of public health staff incorporating cultural safety knowledge into their daily practice. We also consulted with public health staff and scholars to determine competencies and indicators for successfully working with Indigenous peoples broadly. Finally, we conducted an environmental scan in June 2021 showing a dearth of information about cultural safety indicators and evaluation of incorporation in organizational settings [16]. The tool was grounded in the following intervention assumptions: (1) staff who increase their knowledge and awareness of Indigenous peoples and cultural safety will increase their motivation to support Indigenous communities through programs, policy, and advocacy; and (2) staff who increase their knowledge, including self-awareness, through completion of the course will increase their confidence and skills in Indigenous cultural safety.

### Talking circle

In accordance with our partnership agreement, a virtual talking circle was held in December 2021 and attended by thirteen health unit managers and senior leaders. A local Elder opened the virtual space with an opening ceremony and the principal investigator facilitated the talking circle. The public health unit participants were asked if they thought the checklist would help them along their path of working with Indigenous peoples. Every staff member in the call was given an opportunity to answer if they desired; three themes emerged in response: uses of the tool, learning, and collaborative approaches.

The tool was described by staff as “helpful”, “flexible”, and as having multiple uses. One manager noted how the tool would help determine what they needed to observe during evaluations to make it relevant to the staff on their team. Staff felt that the use of the tool extended beyond an evaluation purpose and included being used as a “platform for conversations” when talking to other staff about their individual learning journeys, being more aware of what needed to be learned individually and adapting existing community engagement resources based on the tool.

Five staff members described their own individual learning journeys and how they still had a lot to learn about Indigenous peoples and the components of the tool itself. One staff member stated that “we’re on a long learning journey” and that “there’s always more”. In response to this, it was proposed by staff that the “completed” column (used to measure each indicator) in the tool should be changed to “integrated into practice”. Other staff stated how they felt like their learning journey had just started and that it would be impossible to evaluate their team members until they learned more. This indicates that staff at all levels of the organization need to develop knowledge and competency so that managers are able to evaluate staff in the future and ensure work and engagements are culturally safe at all levels of the organization.

Using the tool for team/organization evaluations instead of individual evaluations were mentioned by three staff members. It was also mentioned that on some teams, most staff worked in isolation, thus it would be difficult to evaluate each staff member individually. Some proposed that the tool be used as a “collective tool”, “learning tool”, or “reflection tool” instead of just an evaluation tool. Another staff member proposed that the tool could be used collaboratively to learn from others as some staff had more experience working with Indigenous people.

## Discussion

Tools for measuring and monitoring staff performance are important to understand strengths and areas for improvement at both individual- and organizational-levels [50]. Furthermore, there are currently no national-level measures or data related to cultural safety [50]. Evaluation tools such as the ICSEC that we developed can be used to address this gap. Incorporating self-reflection, history, knowledge, and skill-based indicators ensure that all aspects of the cultural safety continuum (awareness, sensitivity, competency, safety) are assessed using the evaluation tool [20]. Additionally, embedding staff performance indicators on cultural competence ensure organizational responsibility to Indigenous health priorities and can be an initial step in answering the Truth & Reconciliation Commission’s Calls to Action [9].

The Royal Commission on Aboriginal People report [32] recommended that Canadians, regardless of residency or occupation, become knowledgeable about First Nations, Inuit, and Métis issues to reduce stigma, racism, and improve relations and move toward Indigenous self-determination and governance. The ICSEC will not provide Indigenous communities with self-determination or governance, but will assist healthcare and public health professionals with evaluating whether staff have the required foundational knowledge for working with First Nations, Inuit and Métis peoples, communities and organizations as they continue to achieve self-determination and governance in health and well-being [51, 52]. In this learning journey of cultural safety, public health and health professionals will become aware of historic and contemporary issues that relate to their employment and personal lives, which can assist them with becoming allies, or be advocates for Indigenous peoples or clients to have true self-determination over their health and well-being [51].

The incorporation of Indigenous worldviews, cultural values, and contextual factors within the ICSEC tool was also important to ensure relevance and community benefits of the tool for local Indigenous people accessing services provided by the public health unit. First Nations communities and urban Indigenous organizations often struggle to access resources, programs or services that are culturally safe and welcoming for Indigenous peoples to use without experiencing racism or discrimination [9, 24, 53]. Therefore, evaluating if cultural safety training is being incorporated or integrated into public health staff daily practice and/or determining future learning required will assist with improving interactions that First Nations, Inuit, and Métis people have with public health and healthcare professionals. The ICSEC tool is only one possible method of determining if public health staff are self-aware and reflective of microaggressions, racism, and stereotypes that make spaces and interactions uncomfortable, at best, or fatal, at worst.

Without accountability tools to ensure the uptake of cultural safety trainings, alongside other institutional interventions, Indigenous peoples in Canada will continue to face discrimination, racism, refusal of service, or neglect, all of which can lead to decreased quality of life impacting the overall health and well-being of Indigenous peoples, communities, Nations, and organizations. The negative, or anything less than positive, experiences that Indigenous peoples face have a ripple effect; intra-generational trauma is not often discussed, but occurs when siblings, cousins, and peers of the same age cohort or generation face harmful interactions. In this case, Indigenous peoples who themselves have experienced racism, discrimination, or harm in interactions with public health or healthcare professionals will likely relay that information to family and friends, which then makes individuals less likely to seek the programs or services they require to improve their health and well-being [54, 55]. Using the ICSEC tool, non-Indigenous organizations will be moving toward accountability to Indigenous peoples, communities and organizations that can truly have effects beyond one Indigenous individual and can assist with many tangentially related issues like self-determination, self-governance, improved health and well-being of individuals, communities and organizations, and improved policies, programs and services that are welcoming and safe for all made-vulnerable peoples. Accountability tools and interventions are important to sustain the long-term effects of cultural safety education and prioritize the wellbeing of Indigenous communities. Our experience can provide guidance to health professionals in creating and measuring the efficacy of Indigenous cultural safety education to foster an anti-racist work culture as well as improved health outcomes among Indigenous communities.

There is a continued need for non-Indigenous health service providers to develop culturally safe environments through policies to appropriately serve Indigenous clients. The environment must also demonstrate a decreased tolerance for socially unacceptable behaviour, such as racism [41]. For example, the public health unit of interest demonstrated a top-down effect in which senior staff deemed Indigenous health priorities as important, in addition to establishing the responsibility of managers in evaluating staff competencies. Policy scholars state that the use of multiple policy tools, like regulation (sticks), economic means (carrots), and information (sermons), are often cost-effective in behaviour change at the individual and organizational levels [41, 56]. Referring to behavioural change theories like the Transtheoretical Model and Social Norms theory can provide insight in understanding the impact of staff performance evaluations towards Indigenous health priorities. According to the Transtheoretical Model of Behavior Change (Stages of Change), people must move through the six stages as we’ve mentioned earlier in this article. Conversely, using the Social Norms theory, individuals commonly engage in socially problematic behaviour like racism and oppression because of their perception that the behavior is more socially accepted by peers and the broader community [41]. The theory also predicts that interventions aimed at exposing the actual norm that centres social cohesion can reduce an individual’s participation in problematic behaviours [41]. These models share similarities to the Indigenous cultural safety continuum, where individuals need to gain more knowledge about Indigenous peoples and engage in relevant activities to move from cultural awareness to cultural safety [23, 38, 39, 57]. In contrast to the western behavioral change theories listed here, the cultural safety continuum explicitly suggests the need for individuals to experience a sense of discomfort. However, it is important to note that some of these theories are context-dependent and may not be relevant to all forms of staff evaluations. Instead, further research on evaluating staff evaluations related to Indigenous cultural safety is required.

### Limitations

We are aware that due to the COVID-19 pandemic and related barriers, limited community consultations were able to be conducted. The purpose of this paper is to provide the context as to the development of the evaluation tool and its use. Since this evaluation tool was developed as a pilot, further research is needed to evaluate the implementation and use of the tool itself.

In alignment with community-based approaches centring the needs of the public health unit, the tool incorporated a western approach to public health. This was desirable by the staff as many of them have little to no knowledge of Indigenous approaches. In designing the evaluation tool, our team and partners found it necessary to first build the foundational knowledge of Indigenous peoples, culture, and people, and then move into more decolonizing work that encompass Indigenous pedagogies and methodologies. As the staff are still in the early stages of their learning journey, a model reflective of conventional western health promotion models was deemed relevant, appropriate, and applicable. We see this evaluation tool as a starting point for organizations, particularly public health units, wishing to operationalize accountability measures into their workplace. There would be significant benefit for another tool to be developed utilizing exclusively Indigenous evaluation frameworks and evaluating staff’s uptake of Indigenous cultural safety from an Indigenous lens.

Indigenous cultural safety is an ongoing practice, not something that itself can be demonstrated solely through an evaluation. Achieving some level of Indigenous cultural safety in any organization requires all levels of organization to develop knowledge and proficiency in this work which this tool can support but the tool alone cannot ensure this work is taken up in a meaningful way. Over time, a workplace can use this tool to monitor the uptake of cultural safety across the institution and use this evaluation to incentivize the development of these competencies. It is important to note that this evaluation tool cannot achieve Indigenous cultural safety without organizational transformation more broadly which involves working in partnership and collaboration with Indigenous stakeholders. The challenges presented by senior managers at the PHU suggest that as many people (staff, managers and leaders) are still learning about culturally safety and how to provide a culturally safe environment when working with Indigenous peoples, the ICSEC tool should be iterative as part of a learning health system.

## Conclusion

The creation of the Indigenous cultural safety evaluation tools has potential to assist public health units in advancing Indigenous health priorities. More research is needed to pilot the evaluation tool and make it available and appropriate for different health settings including primary healthcare practices. This is not an area of research our team is currently undertaking but is an appropriate and important avenue for further work on this topic. Any evaluation tool must be iterative to ensure that it is meeting the needs of the local First Nations, Métis and Inuit communities and organizations as well as the needs of the organization is intending to assist in becoming culturally safe; the ICSEC tool is a basic tool that must be adapted by users who are in true and authentic relationships with Indigenous peoples, communities and organizations to ensure that the cultural safety training is being incorporated and practiced by staff, managers and leaders within the organization. By learning about Indigenous peoples, the role of public health staff, and partnership building, staff can be equipped with the knowledge and strategies in fostering culturally safe and relevant spaces for optimizing health care delivery. The ICSEC tool can be used by organizations to reinforce commitments and enhance accountability for the use of cultural safety knowledge in daily interactions that public health staff have. The checklist can be revised to be used in annual performance reviews for employees in different settings and continue to be a living document as the organization changes and social justice is incorporated into the operations of the organization.

### Implications for practice and policy

Accountability tools are important to sustain the long-term effects of Indigenous cultural safety education and prioritize the rights and wellbeing of Indigenous peoples and communities. Our experience can provide guidance to health professionals, specifically public health professionals, in creating and measuring the efficacy and practice of Indigenous cultural safety education to foster an anti-racist work culture as well as improving health outcomes among Indigenous peoples and communities. The ICSEC, or similar tools, analyzing the incorporation of anti-racism and anti-oppression knowledge, skills, and use can be part of policies at all health organizations to move toward an equitable provision of care.

## Electronic supplementary material

Below is the link to the electronic supplementary material.

Supplementary Material 1

## Data Availability

The datasets used and/or analysed during the current study available from the corresponding author on reasonable request. This follows OCAP® principles of First Nations research in Canada.

## Abbreviations

ICSEC: Indigenous Cultural Safety Evaluation Checklist
TRC: Truth & Reconciliation Commission (Canada)
