# Supplementary material for: Designing accountability measures for health professionals: results from a community-based micro-credential: case study on Indigenous cultural safety
Source: BMC Public Health. 2023 May 12;23:879. doi: 10.1186/s12889-023-15721-9 (PMC10176280; doi:10.1186/s12889-023-15721-9)
Supplement: Supplementary file 1 — Supplementary Material 1 [file 12889_2023_15721_MOESM1_ESM.docx]

# Indigenous Cultural Safety Evaluation Checklist for Cultural Safety Micro Credential

Developed by [authors]

This evaluation offers an overview of competencies relevant for the staff of [name] Public Health to demonstrate cultural safety when interacting with Indigenous Peoples. As [name] Public Health take the Indigenous Cultural Safety micro credential course and develop their cultural safety skills and knowledge through their work, staff members will increase their knowledge of Indigenous health terminology, cultural protocols, Indigenous ways of knowing and worldviews, self-awareness and reflexivity, and awareness as to the inter- and intra-generational effects of historical and ongoing colonization. This evaluation was created using two underlying understandings; the first is that as staff members deepen their understandings of cultural safety and their motivation to support Indigenous communities through programs, policy, and advocacy will deepen. The second understanding used to design this evaluation checklist is that staff will increase their confidence and skills in Indigenous health and Indigenous cultural safety through the completion of the cultural safety micro credential.

We also understand and acknowledge that the micro credential cannot offer staff members all the knowledge, skills, and experiences needed to become culturally safe in working with Indigenous Peoples. The course is designed to introduce staff members to the concepts of Indigenous cultural safety and the diversity, needs, and histories of Indigenous Peoples in the Region to support them as staff members continue their work towards working in culturally safe ways. We also suggest implementing reflective journals into regular staff training, meetings, and retreats for an overall enhanced experience.

Date: ________________________________ Time: _____________

Staff name: ________________________________ Position: ________________________________

Evaluator’s name: ________________________________ Position: ________________________________

This checklist evaluates the performance of [name] Public Health staff members who have taken cultural safety micro credential course. Results of this checklist will be used on a routine basis to monitor professional development among staff and to inform staff and managers of their next steps. After completing the cultural safety micro credential course, staff members will enhance cultural competencies, including knowledge, motivation, and self-confidence, in delivering culturally safe care.

There are five components of this checklist that assess changes in a staff member’s cultural competencies as it relates to their job responsibilities:

- - Terminology
    - This component assesses the staff member’s knowledge about appropriate use of Indigenous health terminology and land acknowledgments.
  - Knowledge
    - This component assesses the staff member’s knowledge of Indigenous cultural protocols and engagement, significance of traditional tobacco, Indigenous worldviews, Indigenous health concepts, and the impacts of colonization,
  - Awareness
    - This component assesses the staff member’s self-awareness of concepts related to their social positionality including power and privilege.
  - Skills
    - This component assesses the staff member’s skills in appropriate, respectful, and confident engagement with Indigenous communities given rising local contexts, as it relates to their job responsibilities.
  - Behaviours
    - This component assesses the staff member’s ability to address community needs, foster long-term relations, and advocate on behalf of Indigenous communities.

## Terminology and Land Acknowledgments

| **1** | **Indicator** | **Not in place** | **In progress** | **Completed/In place** | **Not applicable** | **Don’t know** | **Notes** | **Overall Score** |
| --- | --- | --- | --- | --- | --- | --- | --- | --- |
| 1.1 | Appropriate use of terminology  The staff member refers to a person as a member of their community/nation or as Indigenous. Stereotyped and outdated terminology such as “Indian” and “Aboriginal” are not used unless referring to historical contexts. |  |  |  |  |  |  |  |
|  | 1. Orally in internal settings |  |  |  |  |  |  |  |
|  | 1. In online internal communications |  |  |  |  |  |  |  |
|  | 1. Orally with Indigenous communities |  |  |  |  |  |  |  |
|  | 1. In online communications with Indigenous communities |  |  |  |  |  |  |  |
| 1.2 | Person-centred language  The staff member uses language that puts the person first rather than their condition. |  |  |  |  |  |  |  |
| 1.3 | Appropriate use of land acknowledgments  The staff member knows how and when to acknowledges the land they are on, the Indigenous nations whose territory it is, and the treaties which govern it. |  |  |  |  |  |  |  |
| 1.4 | Connection to the land  The staff member is intentional in delivering a land acknowledgment by connecting it to their social positionality. |  |  |  |  |  |  |  |

## Knowledge

| **2** | **Indicator** | **Not in place** | **In progress** | **Completed/in place** | **Not applicable** | **Don’t know** | **Notes** | **Overall Score** |
| --- | --- | --- | --- | --- | --- | --- | --- | --- |
| 2.1 | Knowledge of traditional tobacco  The staff member demonstrates an understanding of traditional tobacco/seema and its significance in Indigenous engagement protocols. |  |  |  |  |  |  |  |
|  | 1. Demonstrates understanding of traditional tobacco and its cultural significance |  |  |  |  |  |  |  |
|  | 1. Demonstrates understanding of when and how to offer tobacco |  |  |  |  |  |  |  |
| 2.2 | Knowledge of engagement protocols  The staff member demonstrates the ability to engage with Indigenous Peoples from a place of respect, reciprocity, and relevance. |  |  |  |  |  |  |  |
| 2.3 | Knowledge of local Indigenous communities  The staff member has engaged with and has demonstrated knowledge of local Indigenous cultural protocols/practices and of local Indigenous communities in the region |  |  |  |  |  |  |  |
| 2.4 | Knowledge of Indigenous worldviews  The staff member demonstrates an understanding of and openness to Indigenous worldviews including the importance of land, and the 4 dimensions of health (physical, mental, emotional, spiritual).  *Note in future years, staff members should demonstrate how they have included Indigenous worldviews in the work that they perform. |  |  |  |  |  |  |  |
| 2.5 | Knowledge of Indigenous health concepts  The staff member demonstrates knowledge about the Medicine Wheel, the web of being or “All My Relations” concepts, and Indigenous social determinants of health.  *In future years, staff members will need to be able to link the Indigenous social determinants of health to the work/programs that they work on. |  |  |  |  |  |  |  |
| 2.6 | Knowledge of impacts on health  The staff member demonstrates an understanding of the impacts of colonization on Indigenous Peoples in the regions. |  |  |  |  |  |  |  |
|  | 1. Has reflected and/or included knowledge of colonization’s impacts on Indigenous Peoples in their work. |  |  |  |  |  |  |  |
|  | 1. Has engaged with and leveraged Indigenous Peoples’ voices as an approach to Indigenous self-determination in their work. |  |  |  |  |  |  |  |

## Awareness

| **3** | **Indicator** | **Not in place** | **In progress** | **Completed/In place** | **Not applicable** | **Don’t know** | **Notes** | **Overall Score** |
| --- | --- | --- | --- | --- | --- | --- | --- | --- |
| 3.1 | Positionality  The staff member demonstrates an ability to recognize and reflect meaningfully on their social positionality in terms of race, class, gender, sexuality, ethnicity, and ability.  *Meaningful reflection and actions refer to the commitment to addressing Indigenous health inequities and focus respectful relevant and useful action-oriented responses. These actions are centred around the client/community and not upon the actor. |  |  |  |  |  |  |  |
| 3.2 | Privilege  The staff member demonstrates an ability to recognize and meaningfully reflect upon how their positionality is associated with special advantages and reduced barriers in attaining certain social and economic goals in the work they do. |  |  |  |  |  |  |  |
| 3.3 | Power  The staff member recognizes their privilege and demonstrates allyship by meaningfully engaging with Indigenous Peoples to set priorities and create concrete change. |  |  |  |  |  |  |  |
| 3.4 | Importance of Relationships  The staff member demonstrates an understanding of relationality of how the development of respectful relationships/partnerships with Indigenous community stakeholders can have a positive influence on health outcomes. |  |  |  |  |  |  |  |
|  | 1. Has built or enhanced authentic and meaningful relationships |  |  |  |  |  |  |  |
|  | 1. Nurtures authentic and meaningful relationships with Indigenous community stakeholders through attending community events, a variety of interactions (telephone, video calls, in-person) to further understand and integrate Indigenous worldviews in their work. |  |  |  |  |  |  |  |
| 3.5 | Self-Reflection  The staff member demonstrates an ability to consider how their own approach, actions, or communication reflects their own cultural perspective/worldview. |  |  |  |  |  |  |  |
|  | 1. Regularly completes self-reflections at organizational retreats and meetings |  |  |  |  |  |  |  |
|  | 1. Regularly engages in self-reflection conversations (orally or written) with internal colleagues |  |  |  |  |  |  |  |

## Skills

| **4** | **Indicator** | **Not in place** | **In progress** | **Completed/in place** | **Not applicable** | **Don’t know** | **Notes** | **Overall Score** |
| --- | --- | --- | --- | --- | --- | --- | --- | --- |
| 4.1 | Contextualize present Indigenous health issues  The staff member has a demonstrated ability to contextualize local Indigenous health issues within the ongoing impacts of colonization and intergenerational trauma. |  |  |  |  |  |  |  |
|  | 1. Integrates Indigenous worldviews into planning, delivery, and/or their work |  |  |  |  |  |  |  |
|  | 1. Integrates Indigenous health concepts into planning, delivery, and/or their work |  |  |  |  |  |  |  |
|  | 1. Has changed the planning, delivery, or general practice of their work to reflect local Indigenous health issues |  |  |  |  |  |  |  |
| 4.2 | Appropriate communication and engagement  The staff has demonstrated the ability to respectfully and confidently engage with community stakeholders while demonstrating awareness of various cultural contexts and Indigenous protocols. |  |  |  |  |  |  |  |
|  | 1. Has included opening and closing ceremonies in meetings led by Elders or Knowledge Keepers. |  |  |  |  |  |  |  |
|  | 1. Interacts with partners in a way that gives space and power to Indigenous partners |  |  |  |  |  |  |  |

## Behaviour

| **5** | **Indicator** | **Not in place** | **In progress** | **Completed/In place** | **Not applicable** | **Don’t know** | **Notes** | **Overall Score** |
| --- | --- | --- | --- | --- | --- | --- | --- | --- |
| 5.1 | Addressing community needs  Staff member provides timely responses when appropriate including information, resource allocation, and referrals. |  |  |  |  |  |  |  |
| 5.2 | Continuous communication and relationship building  Staff member is in relationship with Indigenous community members. |  |  |  |  |  |  |  |
|  | 1. Engages in active listening without interrupting and reflective listening by paraphrasing what is said by Indigenous clients or community members. |  |  |  |  |  |  |  |
|  | 1. Staff member contributes comments and uses language that affirms Indigenous lived experiences and upholds Indigenous worldviews, knowledges, and values. |  |  |  |  |  |  |  |
|  | 1. Has regular communication with Indigenous partners about major public health decisions, updates, and changes. |  |  |  |  |  |  |  |
|  | 1. Has regular open, public community consultations, as defined by the community, about major public health decisions, updates, and changes. |  |  |  |  |  |  |  |
|  | 1. Seeks ways to maintain ongoing communication (telephone, video calls, in-person) that are most accessible for clients/communities |  |  |  |  |  |  |  |
| 5.3 | Advocacy  Staff member is equipped to advocate as required for Indigenous community member and can accomplish this while respecting the autonomy of the Indigenous community member(s). |  |  |  |  |  |  |  |
| 5.4 | Partnership  Staff member completes their work in partnership with Indigenous communities, as appropriate. |  |  |  |  |  |  |  |
|  | 1. Has established and/or continues to follow existing responsibilities in partnership agreements, Memorandum of Understanding, or an agreement of similar effect. |  |  |  |  |  |  |  |
|  | 1. Has involved Indigenous partners and stakeholders in high-level decision-making processes |  |  |  |  |  |  |  |
|  | 1. Partnerships overseen by staff member ensure Indigenous community members and project partners as co-creators as appropriate. |  |  |  |  |  |  |  |
